# The Pleiotropic Effects of Simvastatin on Retinal Microvascular Endothelium Has Important Implications for Ischaemic Retinopathies

**DOI:** 10.1371/journal.pone.0002584

**Published:** 2008-07-09

**Authors:** Reinhold J. Medina, Christina L. O'Neill, Adrian B. Devine, Tom A. Gardiner, Alan W. Stitt

**Affiliations:** Centre for Vision Science, Queen's University Belfast, Belfast, United Kingdom; University of Oldenburg, Germany

## Abstract

**Background:**

Current guidelines encourage the use of statins to reduce the risk of cardiovascular disease in diabetic patients; however the impact of these drugs on diabetic retinopathy is not well defined. Moreover, pleiotropic effects of statins on the highly specialised retinal microvascular endothelium remain largely unknown. The objective of this study was to investigate the effects of clinically relevant concentrations of simvastatin on retinal endothelium *in vitro* and *in vivo*.

**Methods and Findings:**

Retinal microvascular endothelial cells (RMECs) were treated with 0.01–10 µM simvastatin and a biphasic dose-related response was observed. Low concentrations enhanced microvascular repair with 0.1 µM simvastatin significantly increasing proliferation (p<0.05), and 0.01 µM simvastatin significantly promoting migration (p<0.05), sprouting (p<0.001), and tubulogenesis (p<0.001). High concentration of simvastatin (10 µM) had the opposite effect, significantly inhibiting proliferation (p<0.01), migration (p<0.01), sprouting (p<0.001), and tubulogenesis (p<0.05). Furthermore, simvastatin concentrations higher than 1 µM induced cell death. The mouse model of oxygen-induced retinopathy was used to investigate the possible effects of simvastatin treatment on ischaemic retinopathy. Low dose simvastatin(0.2 mg/Kg) promoted retinal microvascular repair in response to ischaemia by promoting intra-retinal re-vascularisation (p<0.01). By contrast, high dose simvastatin(20 mg/Kg) significantly prevented re-vascularisation (p<0.01) and concomitantly increased pathological neovascularisation (p<0.01). We also demonstrated that the pro-vascular repair mechanism of simvastatin involves VEGF stimulation, Akt phosphorylation, and nitric oxide production; and the anti-vascular repair mechanism is driven by marked intracellular cholesterol depletion and related disorganisation of key intracellular structures.

**Conclusions:**

A beneficial effect of low-dose simvastatin on ischaemic retinopathy is linked to angiogenic repair reducing ischaemia, thereby preventing pathological neovascularisation. High-dose simvastatin may be harmful by inhibiting reparative processes and inducing premature death of retinal microvascular endothelium which increases ischaemia-induced neovascular pathology. Statin dosage should be judiciously monitored in patients who are diabetic or are at risk of developing other forms of proliferative retinopathy.

## Introduction

Statins are potent and effective inhibitors of cholesterol biosynthesis that are widely used to treat hypercholesterolemia. Beyond this well-defined mode of action for statins, several clinical trials such as 4S [1], WOSCOPS [2], CARE [3], and HPS [4] have demonstrated that this class of drugs can protect against cardiovascular disease (CVD) through an additional mechanism that is independent of cholesterol lowering [5]. Guidelines from the UK National Institute for Health and Clinical Excellence (NICE) recommend statin therapy for primary prevention of CVD in adults who have a 20% or greater 10-year risk [6]. A recent meta-analysis of 14 randomised trials demonstrated benefits of statin therapy to reduce vascular mortality in diabetic patients [7]. Consequently, millions of diabetic people are receiving statins [8] despite the fact that their local effects on certain tissues like the retina remain largely unknown.

Diabetic retinopathy (DR) is the most common microvascular complication of diabetes, and it remains a major cause of blindness and visual disability [9]. The non-proliferative phase of DR is typified by progressive vasodegeneration [10] leading to inner retinal ischaemia. As diabetes progresses, reparative function in the retinal microvasculature is significantly impaired [11] and ischaemic hypoxia drives up-regulation of angiogenic growth factors such as vascular endothelial growth factor (VEGF) that eventually promote a pathologic neovascular response. Retinal neovascularisation is accompanied by the formation of contractile scar tissue at the vitreo-retinal interface leading to tractional retinal detachment or vitreous haemorrhage, both resulting in severe visual loss. Extensive breakdown of the blood retinal barrier in response to ischaemia-induced VEGF expression causes diabetic macular oedema, which is also associated with retinal vasodegeneration and constitutes another sight-threatening endpoint in DR [12].

Simvastatin is a commonly used drug of its class that has been shown to improve diabetes-induced coronary endothelial dysfunction [13]. Similarly, a clinical trial has indicated that simvastatin may retard the progression of retinopathy in diabetic patients [14] although a recent case-control study found no association between statin use and the risk of developing DR [15]. Therefore the efficacy of statin therapy for DR is ill-defined, and although there is a potential utility for these drugs to prevent this important diabetic complication, the precise mechanism of action requires further investigation.

The actions of statins are pleiotropic and some appear contradictory. For example, these drugs promote reparative angiogenesis in murine hind limb ischaemia models [16] but may also interfere with new blood vessel growth by inhibiting the geranylgeranylation of RhoA [17]. Furthermore, it has been shown that statins have a biphasic effect on endothelial cell migration, promoting the response at low concentrations, and inhibiting it at high concentrations [18]. The pleiotropic nature of statin action has been demonstrated on various macro and microvascular endothelial cells although less attention has been paid to the highly specialised retinal microvascular endothelium. This is important because the effects of statins differ according to the capillary bed [19] and could have an unforeseen biphasic outcome in the retina, which could be either beneficial or harmful, especially in the context of DR. This study has investigated the effects of clinically relevant concentrations of simvastatin on retinal microvascular endothelial cell function *in vitro*. Additionally, a pre-clinical model of ischaemic retinopathy was used to evaluate the effects of high- and low- dose simvastatin *in vivo*.

## Results

### Biphasic Effects of High- and Low- Concentration Simvastatin on Retinal Microvascular Endothelial Cell Growth

**Figure 1:**
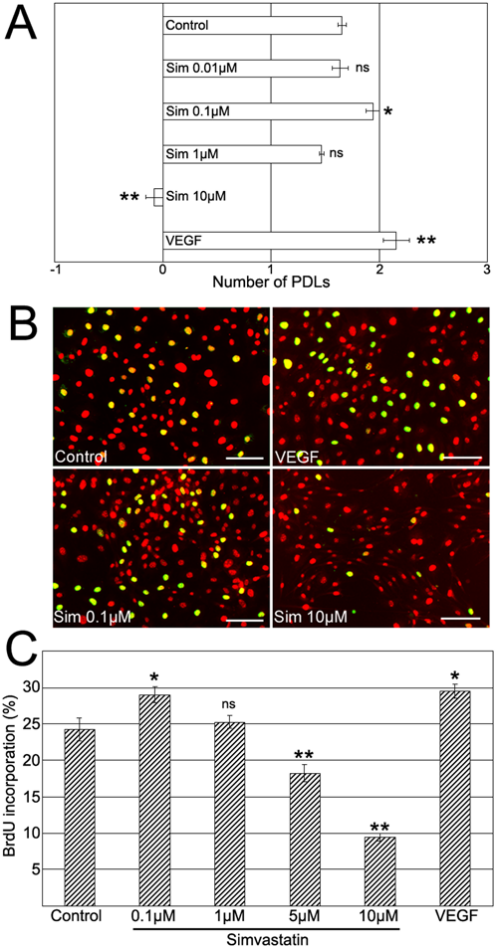
Dose-Dependent Effect of Simvastatin on RMEC Proliferation. (A) RMECs were exposed to 0.01–10 µM simvastatin for 48 hours and the population doubling levels (PDLs) were determined for each treatment-group and compared to controls. *p<0.05, **p<0.01, ns:not significant, n = 4 per group. (B) Immunostaining for BrdU on RMECs exposed to simvastatin (green) identifies proliferating cells, all nuclei stained with PI (red). Scale bars 100 µm. (C) Quantification of BrdU incorporation on RMECs exposed to simvastatin. *p<0.05, **p<0.01 vs. controls, ns:not significant, n = 4 per group.

Retinal microvascular endothelial cells (RMECs) were exposed to concentrations of simvastatin ranging between 0.1–10 µM. Cells treated with 0.1 µM simvastatin demonstrated significantly more population doublings (PD) in 48 hours when compared to controls (1.94±0.06 PD versus 1.66±0.04 respectively). This effect was comparable to that produced by 50 ng/ml VEGF (2.16±0.12 PD in the same time frame). By contrast, 10 µM simvastatin fully suppressed cell proliferation (−0.08±0.08 PD) (Figure 1A). To corroborate these findings we used the Bromodeoxyuridine (BrdU) proliferation assay that identifies replicating cells by their ability to incorporate BrdU into their DNA during the S phase of the cell cycle. The percentage of BrdU-positive cells decreased in a dose-dependent manner (Figure 1C). 0.1 µM simvastatin significantly increased the number of BrdU-positive cells which was akin to the VEGF-induced response (Figure 1B) while 5 and 10 µM simvastatin significantly decreased the number of BrdU-positive cells (Figure 1B). Further analysis of the cell cycle using the PI staining method combined with flow cytometry confirmed that the percentage of RMECs in S phase increased by exposure to 0.1 µM simvastatin, whereas it decreased when cells were exposed to 10 µM simvastatin (Table 1). Therefore, 0.1 µM simvastatin increased RMEC proliferation, while 10 µM inhibited it.

**Table 1:** Cell Cycle Analysis of RMECs by flow cytometry.

		Phase of the Cell Cycle		
	SubG1	G0/G1	S	G2/M
Control	7%	64%	18%	11%
0.1 μM Sim	3%	62%	24%	11%
1 μM Sim	3%	60%	21%	16%
10 μM Sim	13%	64%	13%	10%
10 μM Sim+Mev	5%	64%	16%	15%

Analysis of Cell Cycle was based on DNA content by Propidium Iodide staining assessed by flow cytometry. The values represent means from a total of 10 000 events per group.

**Figure 2:**
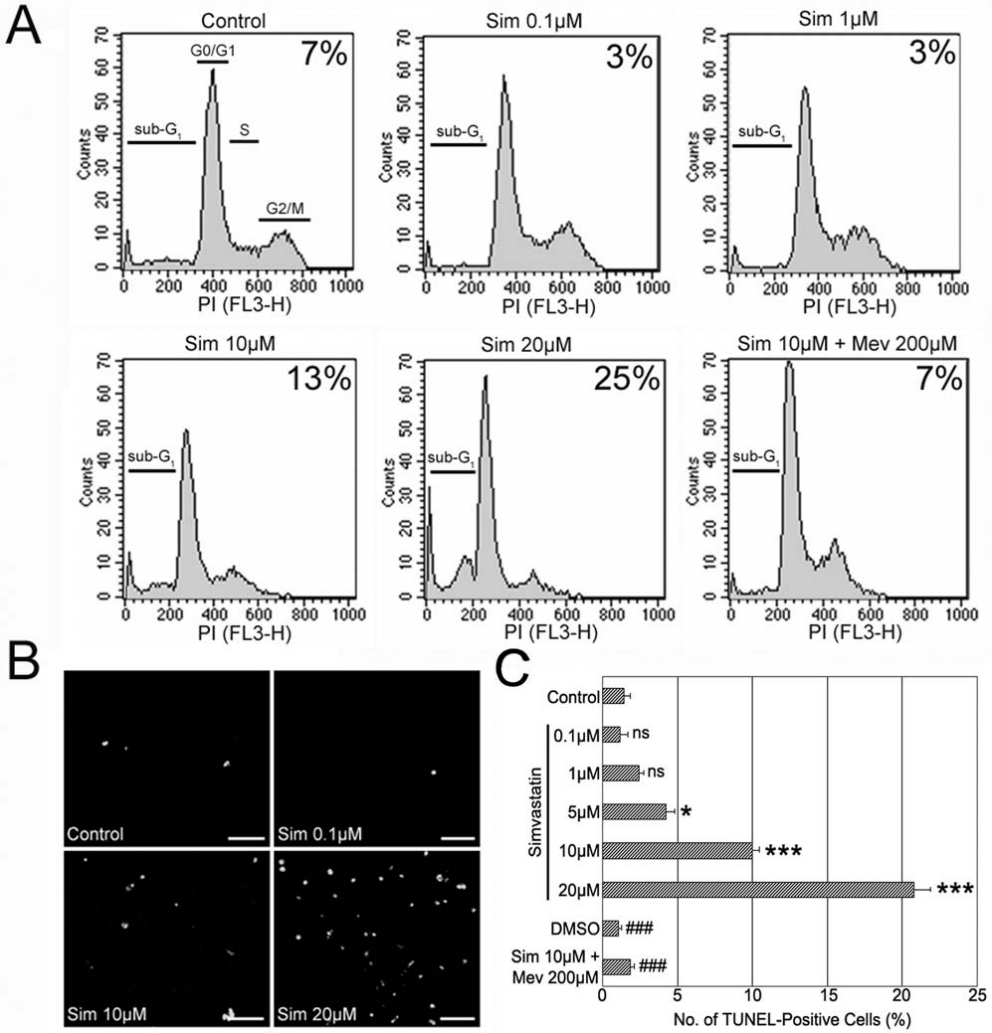
High-Dose Simvastatin Induces Cell Death on RMECs. (A) Analysis of cell death using PI staining and flow cytometry on RMECs after 24 hour-exposure to simvastatin. The number on the top-right corner of each treatment represents the percentage of dead cells (sub-G1 population). (B) Immunocytochemistry for TUNEL to identify dead cells after 24 hour- simvastatin treatment. Scale bars 100 µm. (C) Quantification of TUNEL-positive cells on RMECs exposed to simvastatin. *p<0.05, ***p<0.001 vs. controls; ###p<0.001 vs. 10 µM simvastatin, ns:not significant, n = 5 per group.

To determine if simvastatin of various concentrations modulated RMEC death, monolayers were exposed to 0.1–20 µM simvastatin for 24 hours, stained with Propidium Iodide (PI) after ethanol fixation, and 10 000 cells were assessed by flow cytometry to identify the sub-G1 peak that represents dead cells (Figure 2A). 10 µM simvastatin significantly increased the number of dead cells approximately by two-fold when compared to controls. Significantly, this statin-induced response could be reversed by the addition of 200 µM mevalonate, the end product of 3-hydroxi-3-methyl-glutaryl-CoA reductase (HMG-CoA reductase) indicating that inhibition of this enzyme by simvastatin was directly responsible for induction of cell death. Isoprenoid addition (10 µM Farnesol or 10 µM Geranylgeranyl pyrophosphate) to 10 µM simvastatin also blocked cell death, but only partially. To confirm these results, the TUNEL in situ cell death detection kit was used. RMECs treated with different concentrations of simvastatin for 24 hours were stained for TUNEL to label DNA fragmentation in apoptotic cells (Figure 2B). Consistent with previous results, 10 µM simvastatin significantly increased the number of TUNEL-positive cells (Figure 2C). This time a five-fold increase in dead cells was detected with 10 µM simvastatin compared to controls; the discrepancy in the results obtained was attributed to the higher sensitivity of the TUNEL assay to detect cells undergoing apoptosis while the PI-stained sub-G1 peak identifies only late apoptotic cells. Once more, mevalonate fully blocked the cell death induced by 10 µM simvastatin. Consistent with this result was the observation that prolonged exposure (3 days) of RMECs to 1 µM simvastatin induced a significant rise in dead cells (data not shown). Throughout, Dimethyl sulfoxide (DMSO), the initial solvent for simvastatin, had no effect inducing cell death at the medium concentration of simvastatin-treated cells. These results demonstrate that while low concentrations of simvastatin increased retinal endothelial cell proliferation, high concentrations inhibited cell proliferation and induced cell death *in vitro*.

### Dual Effects of Simvastatin on Retinal Endothelial Cell Migration

**Figure 3:**
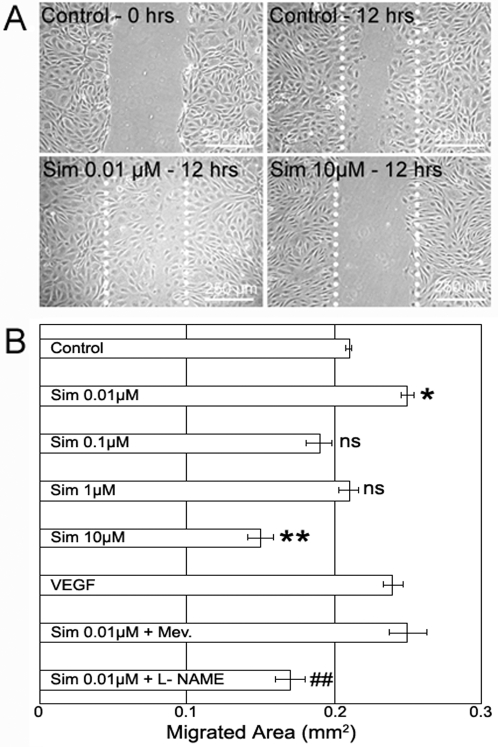
Evaluation of Simvastatin Effect on RMEC Migration. (A) Representative images of the scratch migration wound assay on RMECs treated with simvastatin. White dotted lines mark the wound edge at time-0. (B) Quantification of migrated area 12 hours after the scratch. *p<0.05, **p<0.01 vs. controls; ##p<0.01 vs. 0.01 µM simvastatin, ns:not significant, n = 4 per group.

Pre-confluent RMEC monolayers were treated with 0.01–10 µM simvastatin. When confluent, a scratch wound was made and the denuded area imaged and measured at time-0. 12 hours later the denuded area was again measured and the migratory activity of the endothelial cells estimated (Figure 3A). 0.01 µM simvastatin significantly increased RMEC migration in the scratch wound assay comparable to the effect of 50 ng/ml VEGF. On the contrary, 10 µM simvastatin significantly decreased RMEC migration (Figure 3B). 0.1 µM and 1 µM simvastatin had no significant effect on RMEC migration. Mevalonate addition to the 0.01 µM simvastatin did not reduce cell migration; however N-omega-nitro-L-arginine methyl ester (L-NAME) addition fully inhibited the pro-migratory effect of low dose simvastatin, indicating the involvement of nitric oxide (NO) in RMEC migration. These data show a hormetic response to simvastatin on RMEC migration, characterised as low-dose stimulation and high-dose inhibition.

### Simvastatin Differentially Modulates *In Vitro* Retinal Microvasculature Angiogenesis

**Figure 4:**
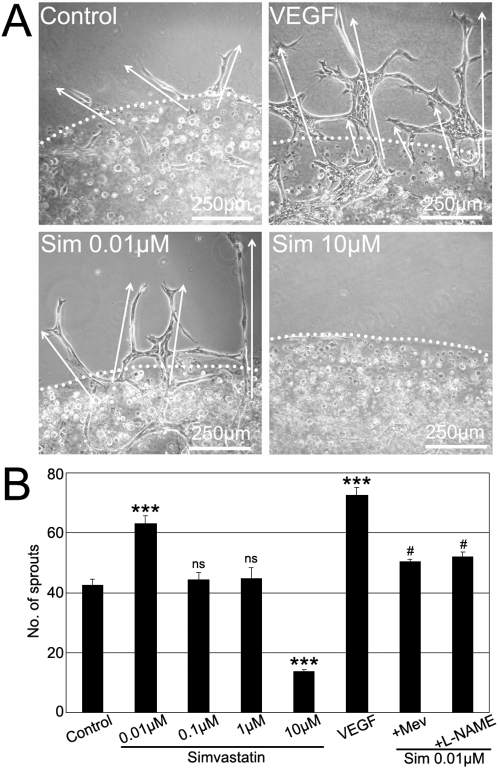
Biphasic Effect of Simvastatin on RMEC Sprouting. (A) Representative phase-contrast images of the sprouting assay on RMECs cultured with 0.01–10 µM simvastatin. White dotted lines represent the boundary between the primary- and secondary-blobs of Matrigel. White arrows mark endothelial sprouts invading the secondary-blob that were quantified for statistical assessment. Note that the shown images are representative fractions (1/12) of the corresponding Matrigel blob-whole circumference. (B) Quantification of the number of sprouts per Matrigel blob. ***p<0.001 vs. controls; #p<0.05 vs. 0.01 µM simvastatin, ns:not significant, n = 3 per group.

Endothelial cell sprouting and tubulogenesis are basic components of the angiogenic process. In order to determine the effects of high- and low- doses of simvastatin on RMEC sprouting and tubulogenesis, respective *in vitro* assays were used. VEGF, as a positive control, significantly promoted RMEC sprout formation by 74% when compared to non-treated RMECs. 0.01 µM simvastatin significantly increased sprout formation by 50%, while 10 µM simvastatin strikingly reduced the number of sprouts per blob by 67% (Figure 4A). The induction of sprout formation by 0.01 µM simvastatin could be partially reversed by co-incubation with mevalonate or L-NAME (Figure 4B).

**Figure 5:**
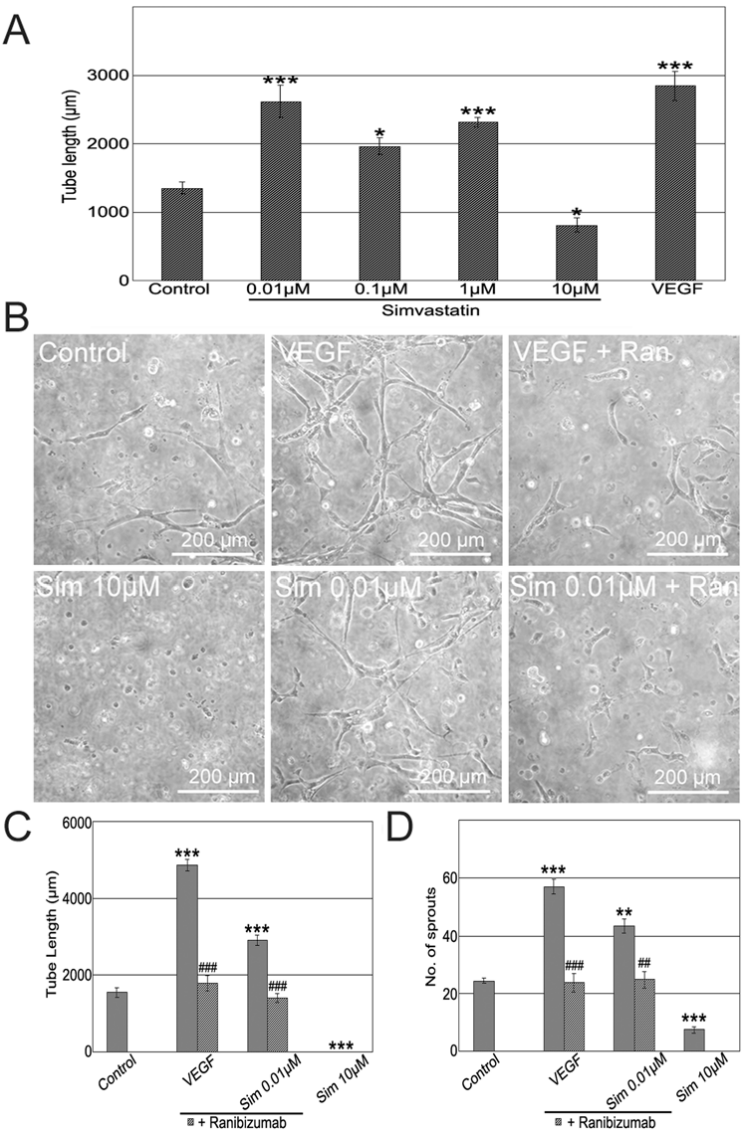
Low-Dose Simvastatin Promotes RMEC Tubulogenesis via VEGF. (A) Quantification of tube length per field to measure and compare tube formation on RMECs exposed to 0.01–10 µM simvastatin. *p<0.05, ***p<0.001, n = 4 per group. (B) Representative images of RMECs cultured on Matrigel and exposed to simvastatin for 3 days. Addition of ranibizumab (Ran) inhibited tube formation on VEGF and 0.01 µM simvastatin groups. (C) Quantification of tube length to demonstrate that 0.1 µM simvastatin effect inducing tubulogenesis involves VEGF. ***p<0.001 vs. control; ###p<0.001 vs. no ranibizumab-respective groups, n = 6 per group. (D) Quantification of sprout number to demonstrate that 0.1 µM simvastatin effect inducing sprouting is mediated by VEGF. ***p<0.001, **p<0.01 vs. control; ###p<0.001, ##p<0.01 vs. no ranibizumab-respective groups, n = 3 per group.

RMECs were cultured on Matrigel extra-cellular matrix to induce tubule formation. Consistent with previous results, 0.01 µM simvastatin significantly promoted tubule formation by 69% when compared to non-treated RMECs, while 10 µM simvastatin inhibited tubulogenesis by 37% (Figure 5A). Moreover, most of the RMECs remained round in matrigel when treated with 10 µM simvastatin (Figure 5B) which suggests an impaired attachment of RMECs to the extracellular matrix when exposed to high dose simvastatin. To investigate whether the simvastatin effect stimulating tubulogenesis was VEGF-dependent, a humanised monoclonal anti-VEGF antibody fragment, ranibizumab, was added to the *in vitro* assays containing 0.01 µM simvastatin. Surprisingly, ranibizumab significantly decreased both tubule formation and the number of sprouts induced by the low simvastatin-concentration to control levels (Figure 5C–D) indicating an important role of VEGF downstream simvastatin. These results indicate that while low-dose simvastatin promotes angiogenesis, high doses impair both sprouting and tubulogenesis of RMECs *in vitro*. The promoting effect of low-dose simvastatin on sprouting and tubule formation is mediated via VEGF.

### Low-Dose Simvastatin Promoted Vascular Repair in a Model of Ischemic Retinopathy

**Figure 6:**
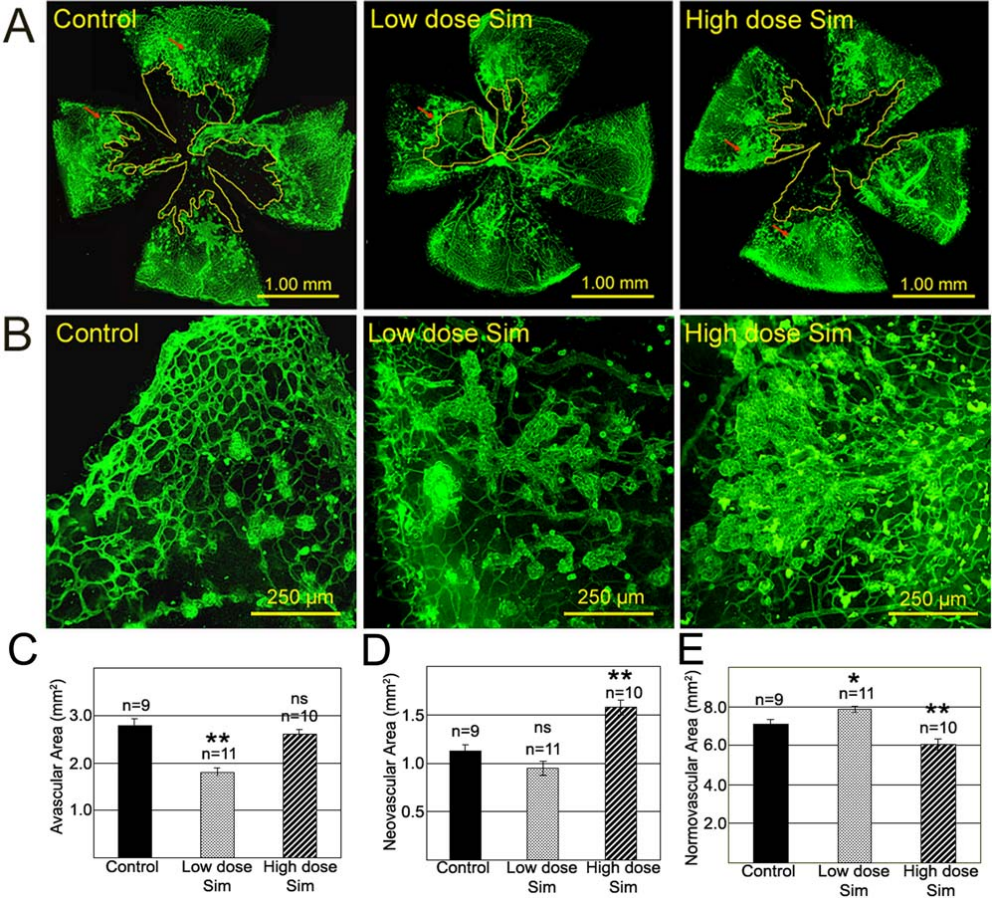
Low-Dose Simvastatin Promotes Vascular Repair in the Ischaemic Retina. (A) Representative images of P18 flat mounted retinas from the oxygen-induced retinopathy murine model. Animals were treated with high- and low-doses of simvastatin from P12 to P17. Lectin staining (green) identifies retinal vasculature, avascular areas are surrounded by yellow lines, and neovascular tufts indicated by red arrows. (B) Higher magnification of lectin-stained flat mounted retinas to show differences in neovascular formations according to the treatment. (C) Quantification of avascular areas per group. **p<0.01 vs. control; ns, not significant. (D) Quantification of neovascular areas per group. **p<0.01 vs. control; ns, not significant. (E) Quantification of normovascular areas per group. **p<0.01,*p<0.05 vs. control; ns, not significant. Error bars in C, D, and E denote standard errors of at least nine independent samples. The statistical significance of differences between controls and simvastatin-treated groups was determined by one-way ANOVA with Dunnett's post-test using GraphPad InStat Version 3.06.

To investigate the possible effects of simvastatin treatment on diabetic retinopathy, the mouse model of oxygen-induced retinopathy (OIR) was used. Mice eyes subjected to this model undergo a reproducible vasodegeneration (P7–12), followed by acute hypoxia (P12–15) leading to aggressive retinal neovascularisation (P15–18) which resembles proliferative diabetic retinopathy. Single daily high or low doses of simvastatin were injected intra-peritoneally from P12 to P17. Flat mounted retinas from P18 mice were assessed for avascular, neovascular, and normovascular areas (Figure 6A–B). Low-dose simvastatin significantly decreased avascular areas by 36% when compared to controls (Figure 6C). High-dose simvastatin significantly increased pathological neovascularisation by 40% (Figure 6D); moreover the neovascular formations were larger and more confluent in the retinas of animals on the high dose regime (Figure 6B). Consistent with these findings, normovascular areas were significantly increased by low-dose simvastatin treatment, and significantly decreased by the high-dose treatment (Figure 6E). These data demonstrate that simvastatin treatment promotes vascular recovery in the ischemic retina, but only when used at a low dose.

### Low Concentration of Simvastatin Induces Akt Phosphorylation and Stimulates Nitric Oxide Production

**Figure 7:**
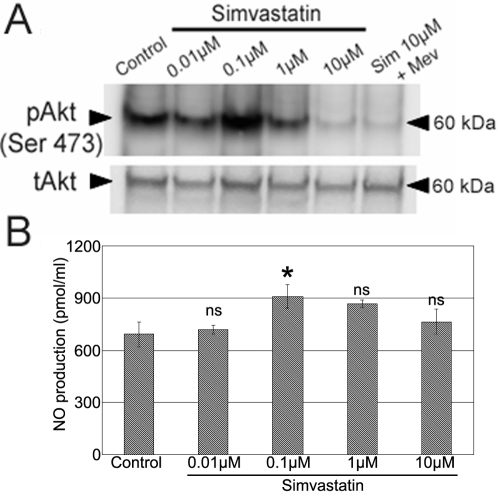
Induction of Akt Phosphorylation and Nitric Oxide Production on RMECs by 0.1 µM Simvastatin. (A) Representative Western blot reveals the effects of simvastatin on Akt phosphorylation depended on the dose. Total Akt (tAkt) remained unchanged. (B) Measurement of nitric oxide production on RMECs exposed to 0.01–10 µM simvastatin for 24 hours using a NO selective microelectrode. *p<0.05 vs. controls, ns:not significant, n = 3 per group.

Previous results demonstrated an important role for VEGF mediating low dose-simvastatin effects (Figure 5C–D) and it is also known that VEGF activates Akt in endothelial cells [20]. In addition, there is evidence that simvastatin activates the protein kinase Akt in human umbilical vein endothelial cells (HUVECs) [16], so the Akt-serine 473 phosphorylation in RMECs treated with different doses of simvastatin was investigated by Western blotting. Increased Akt phosphorylation was detected with 0.1 µM simvastatin (Figure 7A), whereas 10 µM simvastatin decreased the amount of Akt phosphorylation. No changes in total Akt were detected with respective simvastatin concentrations.

Because endothelial Nitric Oxide Synthase (eNOS) is known to be an Akt substrate [21], nitric oxide (NO) production was studied in RMECs treated with simvastatin. NO production was significantly increased by 31% in RMECs treated with 0.1 µM simvastatin when compared to controls (Figure 7B) which is consistent with Akt activation at the same simvastatin concentration. Higher concentrations of simvastatin had no significant effect on NO production. These data provide an insight into possible mechanisms that explain the pro-vascular repair effects of low doses of simvastatin in RMECs, such as Akt activation and NO production.

### High Concentration of Simvastatin Causes Depletion of Intracellular Cholesterol and Disorganization of Actin which Alters Cell Polarisation during Migration

**Figure 8:**
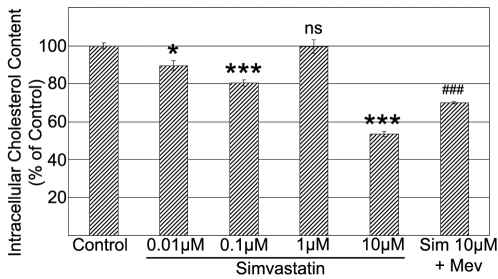
Depletion of Intracellular Cholesterol on RMECs by High-Dose Simvastatin. Determination of intracellular cholesterol content on RMECs after exposure to 0.01–10 µM simvastatin for 24 hours using the Amplex Red Cholesterol Assay. ***p<0.001,*p<0.05 vs. control; ###p<0.001 vs. 10 µM simvastatin, ns:not significant, n = 4 per group.

Here, we observed that the effect of high dose simvastatin on inducing cell death was highly dependent on cholesterol biosynthesis because mevalonate fully reversed the pro-apoptotic effects of high-dose simvastatin (Figure 2C), indicating that intracellular cholesterol metabolism is very important for cell survival. Therefore we assessed the effect of different doses of simvastatin on intracellular cholesterol levels using a quantitative fluorimetric method. 10 µM simvastatin significantly decreased intracellular cholesterol in RMECs by 47% when compared to controls (Figure 8). This drastic intracellular cholesterol reduction corresponds to the cell death induction by 10 µM simvastatin. Lower simvastatin concentrations also reduced intracellular cholesterol levels, but to a minor extent, by less than 20%.

**Figure 9:**
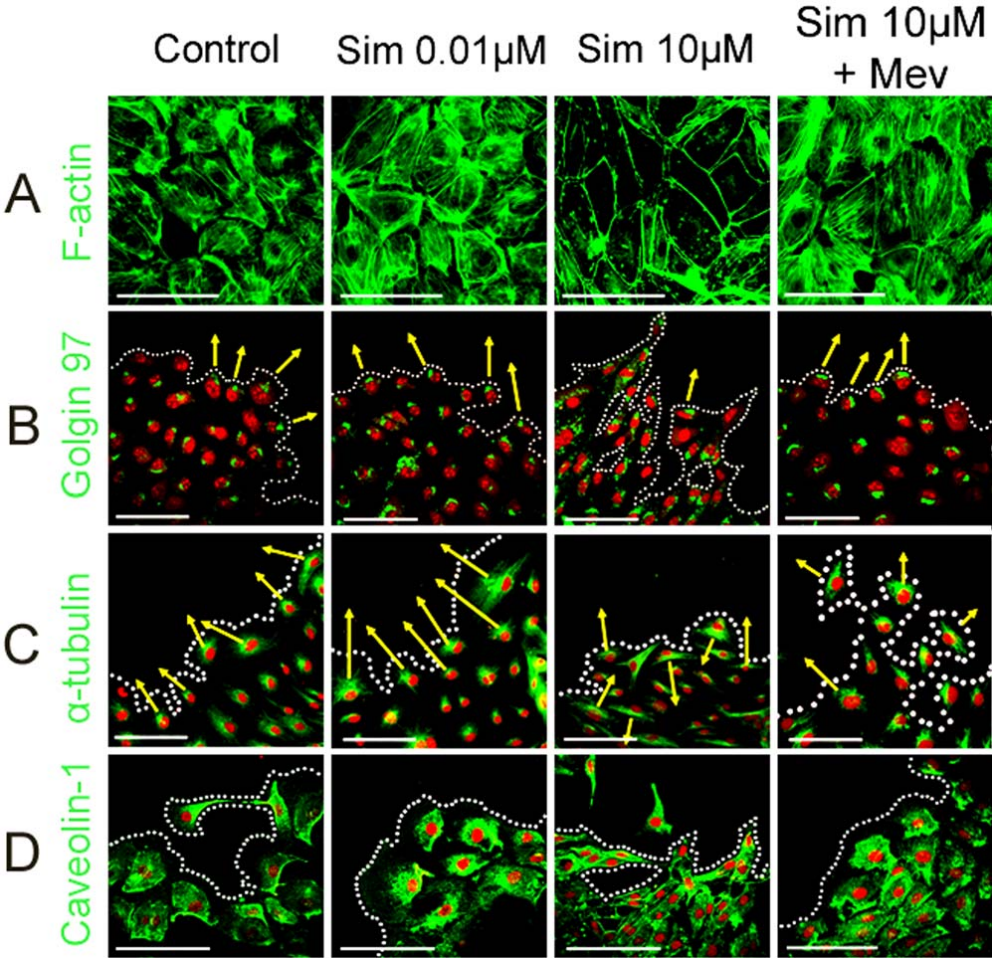
High-Dose Simvastatin Reduces Stress Fiber Formation and Alters RMEC Polarisation during Migration. (A) Immunocytochemistry for F-actin in green using the FITC-phalloidin probe. Scale bars 100 µm. (B) Labelling of the Golgi apparatus on the leading edge of migrating RMECs by staning for Golgin 97 in green. Nuclei are stained in red with PI. Scale bars 100 µm. (C) Identification of the MTOC on the leading edge of migrating RMECs by staning for α-tubulin in green. Nuclei are stained in red with PI. Scale bars 100 µm. (D) Accumulation of caveolin-1 on the trailing edge of migrating RMECs by immunocytochemistry in green. Nuclei are stained in red with PI. Scale bars 100 µm.

Simvastatin can attenuate basal stress fiber formation on pulmonary artery endothelial cells by reducing phosphorylation of myosin light chains [22]. Stress fibers are actin filaments whose formation and remodelling is essential for cell migration, and our results indicated that 10 µM simvastatin inhibits RMEC migration. Therefore we addressed the question whether stress fiber organization in RMECs could be disrupted by simvastatin. RMEC monolayers showed abundant stress fibers. Although 0.01 µM simvastatin did not affect stress fiber formation, 10 µM simvastatin significantly reduced stress fiber formation (Figure 9A). 12 hour-exposure of 10 µM simvastatin to RMEC monolayers resulted in a complete disorganization of actin fibers, an effect that can be reversed by the addition of mevalonate.

To further characterise the effect of simvastatin on RMEC migration, cellular polarisation of the Golgi apparatus, microtubule organisation centre (MTOC), and caveolin was studied in the scratch wound assay using immunocytochemical staining. When RMECs migrated, lamellipodia were formed at the leading edge of the plasma membrane and the Golgi apparatus and MTOC were similarly polarised to the aspect of the nucleus facing the leading edge. The Golgi apparatus identified by the staining of the protein Golgin 97 was polarised toward the leading edge in 73% of the migrating cells (Figure 9B). 0.01 µM simvastatin-treated cells also showed a polarised Golgi in 74% of migrating cells; however 10 µM simvastatin treatment significantly reduced organelle polarisation with only 21% of cells presenting a Golgi polarised towards the wound. Mevalonate could partially reverse this effect and increased the percentage of RMECs with a polarised Golgi to 49% (Table 2). Polarisation of the MTOC on migrating RMECs was also examined (Figure 9C): MTOC, defined as the region from which most microtubules stained with α-tubulin were seen to arise, was quantified as being polarised when facing the wound scratch. Resembling previous results for Golgi polarisation, 10 µM simvastatin significantly decreased the percentage of cells with a polarised MTOC, and mevalonate could partially reverse this effect (Table 2). A role for caveolae in cell migration has also been described [23], so localization of caveolae in the migrating RMEC was identified by immunocytochemistry for caveolin-1. In contrast to the Golgi apparatus and the MTOC polarisation at the leading edge, caveolin-1 was found to accumulate in the rear of most migrating cells as recently reported [24]. 0.01 µM simvastatin-treated RMECs showed the same polarisation pattern for caveolin-1 in the trailing edge, on the contrary there was no specific polarisation for caveolin-1 when RMECs were treated with 10 µM simvastatin (Figure 9D) with caveolin-1 evenly distributed throughout the plasma membrane, both in the leading and trailing edge of the cells. This effect could also be reversed by the addition of mevalonate.

**Table 2:** Percentage of cells with polarised morphology.

	Control	0.01 µM Sim	10 µM Sim	10 µM Sim+Mev
Golgi polarisation	73±3%	74±8%	21±4% ***	49±8% ### (n = 8)
MTOC polarisation	77±2%	86±2%	48±4% ***	66±5% ## (n = 3)

Immunocytochemistry for the proteins Golgin 97 and α-tubulin were used to identify the Golgi apparatus and MTOC, respectively. Percentage of endothelial cells with the Golgi apparatus/MTOC polarised to the leading edge in the Wound Scratch Migration Assay were quantified. All results are presented as mean±SD. ^***^p<0.001 vs. Control; ###p<0.001 vs. 10 µM Sim; ##p<0.01 vs. 10 µM Sim.

These experiments revealed evidence to suggest that high-dose simvastatin induction of cell death is linked to an acute decrease in intracellular cholesterol and a complete disorganization of stress fibers. The same mechanisms may also explain the inhibition of RMEC migration by high- dose simvastatin which is also associated with an impairment of the typical cell polarisation of the Golgi apparatus and MTOC at the leading edge, and caveolin at the trailing edge.

## Discussion

Statins are ubiquitously used throughout the Western world as a therapeutic strategy to control hypercholesterolaemia [25] and thus reduce cardiovascular risk [26]. In some instances, these drugs have been shown to protect against retinopathic progression in diabetic patients and although this may be linked to correction of dyslipidaemia [27], which is a known DR risk factor, it remains possible that statins could directly impact the retinal microvasculature. The current study has evaluated key components of retinal microvascular pathophysiology, particularly focusing on the capillary endothelium since these cells are highly sensitive to the diabetic milieu and are central to DR pathology [28]. Using data obtained from complementary *in vitro* and *in vivo* analysis, we demonstrate, for the first time, that simvastatin has a clear pleiotropic effect on the retinal capillary endothelium.

The dose range used in the current investigation has been carefully chosen to reflect statin use in the clinical situation [29]. However, direct extrapolation of these results to tissue levels experienced by the retina of patients has not been possible. Nevertheless, pharmacokinetic studies have previously demonstrated that serum levels of simvastatin range between 5–30 ng/ml (0.01–0.07 µM) following a single dose of 40 mg (approximately 0.6 mg/Kg) [30]. It can be assumed that the retinal microvasculature is exposed at least to these concentrations *in vivo* and it should be appreciated that simvastatin is prescribed at “for-life” daily doses. Therefore, it seems reasonable to suggest that therapeutic regimes of high-dose statin could lead to considerably elevated tissue accumulations of the drug, especially when considering the lipophilic nature of these compounds.

Previous reports showed that statins have a profound effect on endothelial cell function [18], [31]. Using these studies as a foundation, we have evaluated the effects of simvastatin on several aspects of reparative retinal angiogenesis. Our results indicate that simvastatin has a biphasic dose-related action on RMECs. Low concentrations were pro-angiogenic with 0.1 µM simvastatin significantly increasing cell proliferation, and 0.01 µM simvastatin significantly promoting cell migration, sprouting, and tubulogenesis. High concentrations of simvastatin (>5 µM) had the opposite effect, inhibiting cell proliferation, migration, sprouting and tubulogenesis. Furthermore it is apparent that simvastatin concentrations higher than 1 µM are able to induce cell death. These results indicate that although RMECs are very different to HUVECs, their biphasic response to simvastatin is similar.

To clearly elucidate simvastatin effect on the retinal vasculature *in vivo*, the murine OIR model was used as an appropriate system to study retinal ischaemia leading to pre-retinal neovascularisation. Although relatively acute in nature and based on neonates, the pathology of OIR enables some reasonable comparisons to the late, ischaemic phase of DR. Our results demonstrate that low dose of simvastatin (0.2 mg/Kg) promotes vascular repair in the ischemic retina and this would make it potentially beneficial for preventing vasodegeneration in the context of DR if introduced at the appropriate disease time-frame. Interestingly, the high dose of simvastatin (20 mg/Kg) decreased normal vascularisation, and increased the pathological neovascularisation when compared to controls. It is possible that because simvastatin treatment was started in the hypoxic phase (P12–P15), the high dose actually retarded vascular recovery and increased the hypoxia which in turn led to enhanced pathological neovascularisation. The potential for high-dose statins to abrogate retinal microvascular repair and thus accelerate vasodegeneration would account for the observed enhancement of ischaemia-induced neovascularisation. This conclusion is supported by the complementary *in vitro* and *in vivo* studies.

Our observed pleiotropic effects of statins on endothelial cells may be linked to multiple, inter-related pathways such as VEGF-induced cell activation, regulation of eNOS, and also key signalling responses such as Akt, Rho and Ras [16], [32], [33]. In the current investigation, the data indicate that important statin-induced responses such as sprouting and tubule formation are modulated by VEGF and that the statin effects can be significantly blocked by VEGF neutralisation. Many reports have linked statin actions on endothelium to VEGF upregulation [34]–[36], and since there was no exogeneous VEGF in our *in vitro* angiogenesis assays, this could indicate an autocrine effect of low-dose simvastatin on RMECs. Further linkage to VEGF can be inferred from the finding that low dose simvastatin induces phosphorylation of Akt which is a critical pathway in endothelial cell survival and angiogenesis [37]. Indeed, the Akt pathway is activated by VEGF [38] and this signalling molecule contributes to endothelial cell survival, growth, proliferation, and migration [39]. In addition, Akt phosphorylation activates eNOS [40] which releases NO that, in turn, stimulates vascular remodelling and angiogenesis. Our data also show that 0.1 µM simvastatin increases NO production in RMECs.

High-dose simvastatin appears to have an opposing effect on the retinal microvascular angiogenic reparative process and significantly inhibits cell migration by decreasing intracellular cholesterol and reducing stress fiber formation. This result is important because endothelial cell migration and stress fiber formation are dependent on Rho activation [41] and statins have been shown to block isoprenylation and thus membrane localisation/ functional activation of Rho [42]. It is interesting to note that high-dose simvastatin causes a significant reduction of intracellular cholesterol levels in retinal microvascular endothelium. When it is considered that cholesterol is a key component of cellular membranes having different roles in cellular viability, cell migration, lipid raft assembly and cell signalling [43], this high-dose statin-induced reduction may explain inhibition of cell migration, sprouting, and healing responses culminating in premature vascular cell death.

Results from the immunocytochemical analysis of RMEC polarisation on the scratch wound migration assay (Figure 9 and Table 2) show a strong correlation between polarisation of the Golgi apparatus (73%) and the MTOC (77%) at the leading edge of non-treated RMECs. When treated with 0.01 µM simvastatin, again the Golgi complex and the MTOC similarly polarised to the leading edge (74% and 86% respectively), however 10 µM simvastatin interfered more with the Golgi polarisation which decreased by 71% when compared to controls, while the MTOC polarisation only decreased by 38%. This finding is important as it demonstrates that lowering intracellular cholesterol levels induces an abnormal Golgi complex polarisation in RMECs. It is well-known that the Golgi complex play an essential role in cholesterol transport from its origin in the endoplasmic reticulum to the plasma membrane [44]; therefore modulating cholesterol levels is more likely to affect the Golgi than the MTOC.

Our data indicate the existence of two distinct mechanisms of action for simvastatin on RMECs: the pro-vascular repair mechanism that involves VEGF stimulation, Akt phosphorylation and NO production, and the anti-vascular repair mechanism driven by intracellular cholesterol depletion and actin disorganisation. When simvastatin is used at low doses, the pro-reparative, angiogenic mechanism promotes vascular integrity. However, at high doses the cholesterol depletion is limiting, overwhelming any pro-vascular repair mechanism. This is consistent with the finding that mevalonate fully reversed cell death-induction by high-dose simvastatin, while it could, at best, only partially block pro-angiogenic effects.

In summary, low-dose simvastatin enhances retinal capillary endothelial cell survival, promotes healing responses and modulates angiogenic repair by reducing ischaemia and thereby preventing pre-retinal neovascularisation. High-dose simvastatin prevents reparative function and induces premature death of the retinal microvascular endothelium, which in vivo, translates into excessive ischaemia-induced neovascular pathology. We have not directly evaluated the effects of statins on the diabetic retina but many of the associated vascular pathologic endpoints have been investigated. More research is warranted but there is a clear indication that statin dosage should be judiciously monitored in patients who are diabetic or are at risk of developing other forms of proliferative retinopathy.

## Materials and Methods

### Cell Culture

Primary RMECs were freshly prepared from bovine eyes according to standard isolation procedures previously described [45]. RMECs were cultured on 1% gelatin coated flasks, with DMEM (PAA Labs GmbH, Pasching, Austria) supplemented with 10% porcine serum (Sigma, Gillenham, UK), 100 µg/ml insulin (Sigma), 1 mg/ml heparin (Sigma), and 500 mg/ml Primocin (Autogen Bioclear Ltd, Wiltshire, UK). Passages 3 to 6 were used for experimentation.

### Reagents

Reagents used were: Simvastatin Sodium Salt from Calbiochem (Merck, Darmstadt, Germany), and N-omega-nitro-L-arginine methyl ester (L-NAME), Mevalonate, farnesol, and geranylgeranyl pyrophosphate from Sigma.

### Cell Proliferation and Bromodeoxyuridine (BrdU) Incorporation Assay

When RMECs monolayers were 50% confluent, the total cell number per flask was assessed using an Improved Neubauer haemocytometer (Agar Scientific, Essex, UK) and simvastatin treatments started. After 48 hours, the number of cells per flask was counted, and the Population Doubling Level (PDL) was calculated.

For monitoring DNA synthesis, cells were pre-treated with 30 µM BrdU (Sigma) for 45 minutes prior to fixation in 99% ethanol at −20°C for 20 minutes. Microwave treatment in 10 mM Citrate buffer pH 6.0 was used to denature DNA for antigen retrieval, and cells were stained using monoclonal mouse anti-BrdU (DAKO UK Ltd, Cambridgeshire, UK) as primary antibody, and Alexa Fluor 488 goat anti-mouse IgG (Molecular Probes, Invitrogen Ltd, Paisley, UK) as secondary antibody and visualised by a fluorescence microscope (Nikon).

### Propidium Iodide (PI) Cell Death Assay and TUNEL Staining

Cell death was analysed 24 hours after simvastatin treatment. RMECs were harvested and fixed in ice cold 70% ethanol. Cells were resuspended at 0.5×10^6^ cells/ml in staining buffer composed of 100 µg/ml RNase-A (Sigma), and 40 µg/ml PI (Sigma) in PBS, and incubated at 37°C for 30 minutes. Cells were washed in PBS twice and analysed using a Flow Cytometer (FACScalibur, BD Biosciences, Oxford, UK). Dead cells were detected on a PI histogram as a hypodiploid peak named sub-G1 population. Cell cycle analysis was performed on the same samples.

DNA fragmentation was visualized by TUNEL technology, using an In Situ Cell Death Detection Kit (Roche, Burgess Hill, UK) following manufacturer instructions. Briefly, after microwave treatment in 0.1 M Citrate buffer pH 6.0, DNA strand breaks were detected by 3′-end labelling with fluorescein-dUTP using a terminal deoxynucleotidyl transferase.

### Scratch Wound Assay

RMECs were grown on 1 cm wells previously labelled with traced lines in order to photograph the same regions. When cells were 90% confluent, simvastatin treatment was started. After 24 hours, a uniform straight scratch was made in the monolayer using a 200 µl pipette tip. After injury, cells were gently washed, the medium was changed, and reference photographs were taken using a phase contrast microscope Eclipse E400 (Nikon, Japan). After 12 hours, photographs were again taken and the endothelial cell migration into the denuded area was quantified by comparing areas immediately after the scratch with the ones taken 12 hours later. Lucia software was used to measure areas in mm^2^.

### Angiogenic Sprouting and Tubulogenesis Assay

An *in vitro* angiogenic sprouting assay was performed as previously described [11]. Briefly, 0.5×10^6^ RMECs were resuspended in 25 µl DMEM with respective simvastatin treatments, and mixed in a 1∶1 ratio with growth factor-reduced Matrigel (BD Biosciences). 50 µl aliquots were spotted onto 4 well plates (NUNC, Roskilde, Germany). After Matrigel polymerisation, blobs were covered in DMEM with 10% porcine serum and different simvastatin concentrations. 24 hours later, medium was aspirated and a second layer of Matrigel was superimposed upon the primary spots creating duplex cultures which were covered with DMEM containing the respective simvastatin treatments. 36 hours after creating the duplex cultures, endothelial sprouts could be easily identified invading the secondary gel layer. The number of endothelial sprouts around the circumference of each spot was quantified in 6 cultures per treatment.

For the assessment of tube-like structures formation, 24 well-plates were coated with a basement membrane matrix preparation: growth factor-reduced Matrigel diluted 1∶2 in DMEM. RMECs were plated at a density of 10^4^ cells/well and incubated in 0.01–10 µM simvastatin at 37°C for 3 days. 50 ng/ml VEGF (R&D Systems, Abingdon, UK) was used as a positive control. To test whether simvastatin effects involved VEGF signalling, a VEGF neutralising Fab fragment ranibizumab (Novartis, Basel, Switzerland) was used at a 1/50 dilution (200 µg/ml). Images were captured using a phase contrast microscope Eclipse E400 (Nikon).

### Immunocytochemistry

RMECs were fixed in 4% paraformaldehyde for 30 minutes at room temperature. After blocking with 5% goat serum in PBS with 0.1% Tween 20 for one hour at room temperature, cells were treated with a primary antibody overnight at 4°C. After washing with PBS, cells were incubated with appropriate secondary antibodies for one hour at room temperature, and observed on a Confocal Fluorescence Scanning Microscope (Olympus BX60 epifluorescence microscope fitted with a BioRad Microradiance Confocal imaging system equipped with argon-ion and green Helium-Neon lasers). Omission of the first antibodies consistently gave no signals confirming absence of non-specific binding of secondary antibodies. The primary antibodies used were anti-bovine α-tubulin (Molecular Probes), anti-golgin-97 (Molecular Probes) and caveolin-1 (BD Biosciences). Respective anti-rabbit and anti-mouse Alexa Fluor 488 IgGs were used as secondary antibodies. For labelling F-actin, the FITC-phalloidin probe (Molecular Probes) was used.

### Oxygen-Induced Retinopathy Model

All experiments were performed in conformity to the ARVO Statement for the Use of Animals in Ophthalmic and Vision Research, and UK Home Office Regulations. Oxygen-Induced Retinopathy (OIR) was induced in C57/Bl6 wild type mice according to the protocol of Smith et al [46]. Briefly, P7 newborn mice and their nursing dams were exposed to 75% oxygen (Pro-Ox 110 Chamber Controller Used, Biospherix, Redfield, NY, USA) for 5 days. At P12, they were transferred back to room air cages. Hyperoxia exposure produces widespread vaso-obliteration in the central retinal capillary beds with profound ischaemic hypoxia upon return to room air. Around P15 the retinal ischaemia initiates an aggressive neovascular response at the interface of the perfused retinal periphery and the ischaemic central capillary beds. The neovascularisation is maximal at P17 and declines thereafter. 22 mice were divided into four groups: Group 1 with four pups as P12 controls to check the central vaso-obliteration following hyperoxia; Groups 2, 3, and 4 contained six pups each; Group 2 received daily intraperitoneal injections of a high dose of simvastatin (20 µg/g/day) for 6 days from P12 to P17; Group 3 received a low dose of simvastatin (0.2 µg/g/day) and Group 4 received no treatment. All intraperitoneal injection volumes were adjusted to 40–50 µl. Pups from groups 2, 3, and 4 were killed at P18 with sodium pentobarbital; the eyes were enucleated and fixed in 4% paraformaldehyde. Retinal flat mounts were stained with isolectin B4 (Sigma) and Streptavidin Alexa Fluor 488 (Molecular Probes). Stained retinas were visualised and imaged using a fluorescence microscope and digital camera (Nikon Eclipse). Avascular, neovascularised, and normally vascularised retinal areas were quantified using Image J software.

### Intracellular Cholesterol Quantification

To measure the intracellular cholesterol content of RMECs, the Amplex Red Cholesterol Assay Kit (Molecular Probes) was used. After 24 hours exposure to different concentrations of simvastatin, RMECs were harvested, counted and 1 million cells per group were lysed in RIPA lysis buffer for later reaction with the Amplex Red Reagent according to the manufacturer's protocol. Fluorescence was measured by a Safire Microplate Reader (Tecan, Reading, UK) using excitation at 530 nm and emission detection at 590 nm.

### Western Blotting

Proteins from simvastatin treated-RMECs and controls were precipitated with 10% trichloroacetic acid, and dissolved in 9 M urea (Sigma), 2 M thiourea (Sigma) and 4% CHAPS (Melford Laboratories,Suffolk, UK). 20 µg of protein were loaded per lane and separated with Tris-HEPES-SDS polyacrylamide gels, transferred to a PVDF membrane, and blocked with 5% Blotto (Santa Cruz Biotechnology, CA) in Tris Buffered Saline with 0.1% Tween-20 (TBST). Total and phosphorylated Akt (Ser 473) were detected with polyclonal antibodies from Cell Signaling Technology (Danvers, MA). After washing with TBST, respective horseradish peroxidase-conjugated secondary antibodies (Santa Cruz Biotechnology) were applied, and the blots were developed using the AutoChemi chemilumininescence imaging system (UVP, Upland, CA). PVDF membranes were re-probed with a monoclonal antibody to GAPDH (IMGENEX, San Diego, CA) as a loading control.

### Electrochemical Detection of Nitric Oxide (NO) Production

Medium from 24 hour simvastatin-treated RMECs was collected and stored at −20°C. NO release was measured using a NO selective microelectrode (ami NO-700, Innovative Instruments Inc, Tampa, FL) and an amplifier inNO meter (Innovative Instruments Inc). Addition of 10 µl sample medium to a 0.1 M H_2_SO_4_ solution with 0.5% KI converted the nitrite in the medium to NO, which was measured using the microelectrode that had been previously calibrated using sodium nitrite standards (250 nM to 3000 nM). NO production was measured as pmol/ml.

### Statistical Analysis

All data are expressed as mean±SEM unless otherwise stated. Statistical significance was evaluated by one-way ANOVA with Dunnett's / Tukey-Kramer's post-test using GraphPad InStat version 3.06 for Windows, GraphPad Software, San Diego, California, USA.

## References

[1] (1994). Randomised trial of cholesterol lowering in 4444 patients with coronary heart disease: the Scandinavian Simvastatin Survival Study (4S).. Lancet.

[2] Shepherd J, Cobbe SM, Ford I, Isles CG, Lorimer AR (1995). Prevention of coronary heart disease with pravastatin in men with hypercholesterolemia. West of Scotland Coronary Prevention Study Group.. N Engl J Med.

[3] Sacks FM, Pfeffer MA, Moye LA, Rouleau JL, Rutherford JD (1996). The effect of pravastatin on coronary events after myocardial infarction in patients with average cholesterol levels. Cholesterol and Recurrent Events Trial investigators.. N Engl J Med.

[4] (2002). MRC/BHF Heart Protection Study of cholesterol lowering with simvastatin in 20,536 high-risk individuals: a randomised placebo-controlled trial.. Lancet.

[5] Bonetti PO, Lerman LO, Napoli C, Lerman A (2003). Statin effects beyond lipid lowering–are they clinically relevant?. Eur Heart J.

[6] NIH (2006). Statins for the prevention of cardiovascular events: Technology Appraisal Guidance 94.

[7] Kearney PM, Blackwell L, Collins R, Keech A, Simes J (2008). Efficacy of cholesterol-lowering therapy in 18,686 people with diabetes in 14 randomised trials of statins: a meta-analysis.. Lancet.

[8] Cheung BM (2008). Statins for people with diabetes.. Lancet.

[9] Watkins PJ (2003). Retinopathy.. Bmj.

[10] Mizutani M, Kern TS, Lorenzi M (1996). Accelerated death of retinal microvascular cells in human and experimental diabetic retinopathy.. J Clin Invest.

[11] Stitt AW, McGoldrick C, Rice-McCaldin A, McCance DR, Glenn JV (2005). Impaired retinal angiogenesis in diabetes: role of advanced glycation end products and galectin-3.. Diabetes.

[12] Frank RN (2004). Diabetic retinopathy.. N Engl J Med.

[13] Tawfik HE, El-Remessy AB, Matragoon S, Ma G, Caldwell RB (2006). Simvastatin improves diabetes-induced coronary endothelial dysfunction.. J Pharmacol Exp Ther.

[14] Sen K, Misra A, Kumar A, Pandey RM (2002). Simvastatin retards progression of retinopathy in diabetic patients with hypercholesterolemia.. Diabetes Res Clin Pract.

[15] Zhang J, McGwin G (2007). Association of statin use with the risk of developing diabetic retinopathy.. Arch Ophthalmol.

[16] Kureishi Y, Luo Z, Shiojima I, Bialik A, Fulton D (2000). The HMG-CoA reductase inhibitor simvastatin activates the protein kinase Akt and promotes angiogenesis in normocholesterolemic animals.. Nat Med.

[17] Park HJ, Kong D, Iruela-Arispe L, Begley U, Tang D (2002). 3-hydroxy-3-methylglutaryl coenzyme A reductase inhibitors interfere with angiogenesis by inhibiting the geranylgeranylation of RhoA.. Circ Res.

[18] Urbich C, Dernbach E, Zeiher AM, Dimmeler S (2002). Double-edged role of statins in angiogenesis signaling.. Circ Res.

[19] Brouet A, Sonveaux P, Dessy C, Moniotte S, Balligand JL (2001). Hsp90 and caveolin are key targets for the proangiogenic nitric oxide-mediated effects of statins.. Circ Res.

[20] Fujio Y, Walsh K (1999). Akt mediates cytoprotection of endothelial cells by vascular endothelial growth factor in an anchorage-dependent manner.. J Biol Chem.

[21] Dimmeler S, Fleming I, Fisslthaler B, Hermann C, Busse R (1999). Activation of nitric oxide synthase in endothelial cells by Akt-dependent phosphorylation.. Nature.

[22] Jacobson JR, Dudek SM, Birukov KG, Ye SQ, Grigoryev DN (2004). Cytoskeletal activation and altered gene expression in endothelial barrier regulation by simvastatin.. Am J Respir Cell Mol Biol.

[23] Navarro A, Anand-Apte B, Parat MO (2004). A role for caveolae in cell migration.. Faseb J.

[24] Parat MO, Anand-Apte B, Fox PL (2003). Differential caveolin-1 polarization in endothelial cells during migration in two and three dimensions.. Mol Biol Cell.

[25] Tobert JA (2003). Lovastatin and beyond: the history of the HMG-CoA reductase inhibitors.. Nat Rev Drug Discov.

[26] Thavendiranathan P, Bagai A, Brookhart MA, Choudhry NK (2006). Primary prevention of cardiovascular diseases with statin therapy: a meta-analysis of randomized controlled trials.. Arch Intern Med.

[27] Gordon B, Chang S, Kavanagh M, Berrocal M, Yannuzzi L (1991). The effects of lipid lowering on diabetic retinopathy.. Am J Ophthalmol.

[28] Badr GA, Tang J, Ismail-Beigi F, Kern TS (2000). Diabetes downregulates GLUT1 expression in the retina and its microvessels but not in the cerebral cortex or its microvessels.. Diabetes.

[29] Moon JC, Bogle RG (2006). Switching statins.. Bmj.

[30] Lilja JJ, Neuvonen M, Neuvonen PJ (2004). Effects of regular consumption of grapefruit juice on the pharmacokinetics of simvastatin.. Br J Clin Pharmacol.

[31] Weis M, Heeschen C, Glassford AJ, Cooke JP (2002). Statins have biphasic effects on angiogenesis.. Circulation.

[32] Rikitake Y, Liao JK (2005). Rho GTPases, statins, and nitric oxide.. Circ Res.

[33] Ii M, Losordo DW (2007). Statins and the endothelium.. Vascul Pharmacol.

[34] Chen SD, Hu CJ, Yang DI, Nassief A, Chen H (2005). Pravastatin attenuates ceramide-induced cytotoxicity in mouse cerebral endothelial cells with HIF-1 activation and VEGF upregulation.. Ann N Y Acad Sci.

[35] Matsuno H, Takei M, Hayashi H, Nakajima K, Ishisaki A (2004). Simvastatin enhances the regeneration of endothelial cells via VEGF secretion in injured arteries.. J Cardiovasc Pharmacol.

[36] Frick M, Dulak J, Cisowski J, Jozkowicz A, Zwick R (2003). Statins differentially regulate vascular endothelial growth factor synthesis in endothelial and vascular smooth muscle cells.. Atherosclerosis.

[37] Shiojima I, Walsh K (2002). Role of Akt signaling in vascular homeostasis and angiogenesis.. Circ Res.

[38] Morales-Ruiz M, Fulton D, Sowa G, Languino LR, Fujio Y (2000). Vascular endothelial growth factor-stimulated actin reorganization and migration of endothelial cells is regulated via the serine/threonine kinase Akt.. Circ Res.

[39] Manning BD, Cantley LC (2007). AKT/PKB signaling: navigating downstream.. Cell.

[40] Fulton D, Gratton JP, McCabe TJ, Fontana J, Fujio Y (1999). Regulation of endothelium-derived nitric oxide production by the protein kinase Akt.. Nature.

[41] van Nieuw Amerongen GP, Koolwijk P, Versteilen A, van Hinsbergh VW (2003). Involvement of RhoA/Rho kinase signaling in VEGF-induced endothelial cell migration and angiogenesis in vitro.. Arterioscler Thromb Vasc Biol.

[42] Wolfrum S, Jensen KS, Liao JK (2003). Endothelium-dependent effects of statins.. Arterioscler Thromb Vasc Biol.

[43] Boukhtouche F, Mariani J, Tedgui A (2004). The “CholesteROR” protective pathway in the vascular system.. Arterioscler Thromb Vasc Biol.

[44] Ikonen E (2006). Mechanisms for cellular cholesterol transport: defects and human disease.. Physiol Rev.

[45] Coleman G, Gardiner TA, Boutaud A, Stitt AW (2007). Recombinant alpha2(IV)NC1 domain of type IV collagen is an effective regulator of retinal capillary endothelial cell proliferation and inhibits pre-retinal neovascularisation.. Graefes Arch Clin Exp Ophthalmol.

[46] Smith LE, Wesolowski E, McLellan A, Kostyk SK, D'Amato R (1994). Oxygen-induced retinopathy in the mouse.. Invest Ophthalmol Vis Sci.

